# A Diversified Spectrometric and Molecular Docking Technique to Biophysical Study of Interaction between Bovine Serum Albumin and Sodium Salt of Risedronic Acid, a Bisphosphonate for Skeletal Disorders

**DOI:** 10.1155/2018/6954951

**Published:** 2018-06-28

**Authors:** M. Manjushree, Hosakere D. Revanasiddappa

**Affiliations:** Department of Chemistry, University of Mysore, Manasagangothri, Mysuru, Karnataka 570 006, India

## Abstract

The binding interaction between bovine serum albumin (BSA) and sodium salt of risedronic acid (RSN) was studied by using the FT-IR (Fourier transform infrared), UV-Vis (ultraviolet–visible), fluorescence (emission and synchronous), CD (circular dichroism) spectrometric, and computational (molecular docking) techniques at 289, 297, and 305 K temperatures with physiological buffer of pH 7.40. The conformational and secondary structural changes observed for BSA from CD spectra and by curve fitting procedure were applied to Fourier self-deconvolution in FT-IR spectra. The formation of a BSA-RSN complex was confirmed from UV-Vis spectroscopy. The static type of quenching shown for RSN to BSA was verified from Stern–Volmer and modified Stern–Volmer equations. The binding constant of order 10^5^ was obtained to be confirming that there exists a strong binding interaction between BSA and RSN. Synchronous fluorescence shows that the microenvironment of tryptophan was altered, not tyrosine of BSA; in addition to this, the distance between tryptophan of BSA and RSN was found out from Forster's theory of nonradiation energy transfer. The interaction between BSA and RSN mainly occurred as a result of hydrogen bonds and van der Waals forces, the process is exothermic and spontaneous, and it was achieved through van 't Hoff equation. This interaction was affected by the presence of biologically active Fe^2+^, Ni^2+^, Ca^2+^, Mg^2+^, and Cd^2+^ ions and was also studied. The subdomain IIIA of BSA involved with RSN interaction was authenticated from molecular docking analysis.

## 1. Introduction

**Figure 1:**
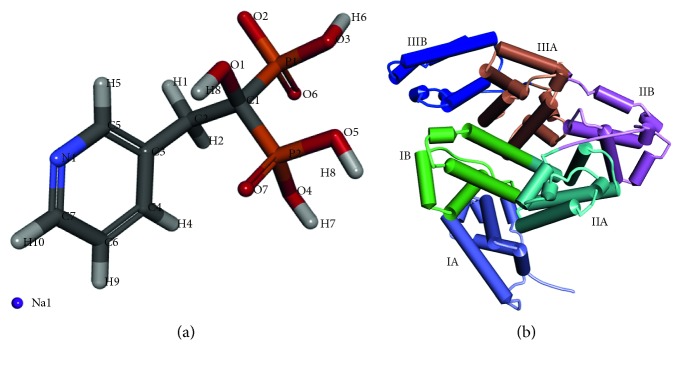
Discovery studio visualization of (a) 3D structure of RSN and (b) secondary structure of BSA. Each site is marked with different colour.

The very most familiar problem arising in the old age of human beings is osteoporosis and Paget's disease [1, 2]. Osteoporosis is the disease that causes breaking of the bone when the bone's weakness increases. This disease mainly affects women compared with men. In 2010 survey, about 22 million women and 5.5 million men in European Union, 8 million women and 1 million men in United states, and Asian people who are at the highest risk were suffered from this disease [3, 4]. Another bone-related issue is Paget's disease which affects one or more bones but not complete skeleton to cellular reshaping and disfigurement such as skull, femur, pelvis, and vertebrae [5]. This disease, on rare cases, converts into severe complication called malignant bone cancer. About 1.5 to 8.0% of British descent [6] have Paget's disease. The ratio of Paget's disease in men to women is of the order 3 : 2 [7]. The well-known drug that is used to treat and prevent osteoporosis and Paget's disease is sodium salt of risedronic acid called as risedronate sodium (Figure 1(a)) which is a bisphosphonate, and its IUPAC name is sodium hydroxyl-(1-hydroxy-1-phosphono-2-pyridin-3-ylethyl)phosphinate. This drug is taken orally as immediate and delayed release tablet [8]. It has common side effects such as back pain, heartburn, diarrhea, and indigestion [9].

In recent years, researchers are focusing more on the interaction between drugs and biomacromolecules [10, 11]. Serum albumin is the most abundant plasma protein present in vertebrate blood. It has a major role in transportation, distribution, and biological process of many endogenous and exogenous ligands (drugs, fatty acids, pharmaceuticals, etc.) to exact targets [12]. The concentration of drug in free and bound form with serum albumin in the plasma gives information on the mechanism of action on the desired disease. Therefore, the rate of evident distribution and elimination of drugs can be found out with help of protein-drug interaction studies. Therefore, this study finds applications in medicinal science for designing drugs and in pharmaceutical industries to improve the drug delivery process.

The present study chooses the bovine serum albumin (BSA) in substitute of human serum albumin (HSA) because of 88% resemblance in structure, easy availability and low cost. The heart-shaped structure of BSA is shown in Figure 1(b) where it is composed of 583 amino acids: whole structure is divided into 3 homologous domains (I, II, and III) and every domains is again subdivided into A and B, with hydrophobic packets with two tryptophan (Trp134 and Trp213) and 20 Tyr (tyrosine) residues. Trp134 and Trp213 are present on the surface and buried inside the hydrophobic region of BSA [13].

The biological features of the protein are then well characterized by essential metal ions binding on to it through their structure and function. In many metalloproteins and enzymes, the metal ions are bound tightly and loosely, respectively, for their stabilization or storage [14–16]. Metal ions with the ratio of charge to ionic radius is called its polarizing power, this is the main factor for complexing power of the metal ions with the proteins. Thus, the increased interaction between BSA and metal ions is achieved by their attaining the high density positive charge in BSA when the polarizibility of metal ions is increased. Metal choices, coordination numbers, and geometries are the main parameters for metal binding sites in BSA. Metal coordinates to BSA are usually through carboxylate (direction of binding is syn lone-pair) group, sulphur groups, and carbonyl oxygens of the main chain, tyrosine, tryptophan, asparagine, serine, aspartic, glutamic, threonine, water molecules, and the side chain of imidazole (lone pairs on nitrogen). Metal ions having unpaired electrons interact more readily with the nuclei of BSA. The native charges and conformational helical dipoles on BSA play an importance role in metal binding. Soft metals bind to sulphur containing residues of methionine and cysteine, while the hard metals bind qualitatively to hydrophilic amino acid residues shell surrounded by hydrophobic (carbon containing) groups of large shell, that is, difference of high hydrophobicity centers [17–19].

The metal ions are very essential for all the living systems where they may affect the binding interaction between serum albumins and drugs. This was also studied in the current work. An insight into the biophysical study of the interaction between BSA and RSN was investigated by employing several spectroscopies (emission fluorescence, synchronous fluorescence, FT-IR, CD, and UV-Vis) and molecular docking methods. Each performed experiment gave characteristic results and their inference is discussed below.

## 2. Experimental

### 2.1. Reagents and Preparation of Solutions

Bovine serum albumin (BSA: product number A1933-1G, chromatographically purified, ≥98%), risedronate sodium (RSN: product number SML0650-50MG, NMR, ≥97%), ammonium iron (II) sulfate hexahydrate (Fe^2+^: product number 203505-5G, trace metal basis, 99.997%), ammonium nickel (II) sulfate (Ni^2+^: product number 574988-25G, trace metal basis, 99.999%), Calcium carbonate (Ca^2+^: product number 202932-5G, trace metal basis, ≥99.995%), magnesium chloride hexahydrate (Mg^2+^: product number 255777-5G, trace metal basis, 99.995%), cadmium sulfate hydrate (Cd^2+^: product number 202924-5G, trace metal basis, ≥99.995%), warfarin (product number A2250-10G, analytical standard), ibuprofen (product number I4883-1G, GC, ≥98%), and digitoxin (product number D5878-250MG, HPLC, ≥92%) were obtained from Sigma-Aldrich (St. Louis, Missouri, USA). A Tris buffer at pH 7.40 (0.05 M Tris and 0.15 M NaCl with few drops of HCl) and double distilled water were used to prepare all the solutions throughout each experiments. The stock solution of BSA at concentration of 1.0 × 10^−4^  mol · L^−1^ was prepared. RSN, warfarin, ibuprofen, digitoxin, and metal ion (Fe^2+^, Ni^2+^, Ca^2+^, Mg^2+^, and Cd^2+^) solutions were prepared as a stock at concentration of 1.0 × 10^−3^mol · L^−1^. For UV-visible, emission fluorescence, and synchronous fluorescence spectroscopic measurements, the concentration of BSA was kept constant (2.5 × 10^−6^mol · L^−1^) by varying the concentrations of RSN from 0.5 to 5.5 (×10^−6^mol · L^−1^) by an increment of 0.5 × 10^−6^mol · L^−1^. All the instrumental spectra were corrected for buffer background.

### 2.2. Methods and Instruments

#### 2.2.1. UV-Visible Spectroscopy

UV-visible absorption spectra in the wavelength range of 200 to 300 nm at 297 K (pH 7.40) were taken using the UV-visible spectrophotometer (Beckman Coulter, DU 730, Life Sciences, Brea, CA 92821, USA). This instrument was equipped with deuterium and tungsten lamps for UV and visible light, respectively. Quartz cuvettes were used with the path lengths of 10 mm.

#### 2.2.2. Fluorescence Spectroscopy

*(1) Instrument*. All the fluorescence spectral measurements were carried out on fluorescence spectrophotometer (F-4600, Hitachi Model, Tokyo, Japan) with 150W Xenon lamp and the excitation and emission slits were kept at 10 nm.

*(2) Inner Filter Effect*. The molecule undergoing reabsorption of radiation emitted results in a noticeable reduction of emission quantum yield at a particular wavelength (emission or excitation) is called inner filter effect. The excitation (295 nm) and emission (∼340 nm) wavelengths of BSA were overlapped with the absorption spectrum of RSN. Therefore, (1) was employed to subtract the fluorescence intensity of RSN from all the BSA fluorescence signals to obtain the desirable fluorescence signals for BSA:

(1) $$\begin{array}{c} F_{\text{cor}}=F_{\text{obs}}\times e^{\left(A_{\text{ex}}+A_{\text{em}}\right)/2}, \end{array}$$

where *A*_ex_ and *A*_em_ are the RSN excitation and emission absorbance of wavelengths, respectively, and *F*_cor_ and *F*_obs_ are the corrected and observed fluorescence intensity of BSA, respectively.

*(3) Fluorescence Emission*. The emission fluorescence spectra were recorded at three different temperatures (289, 297, and 305 K). The required temperatures were maintained by using outer circulating water bath. The excitation wavelength was set to 295 nm, and emission wavelengths were varied from 290 to 420 nm.

*(4) Synchronous Fluorescence*. By setting the Δ*λ* (Δ*λ*=*λ*_em_ − *λ*_ex_) at 15 and 60 nm corresponding to Tyr and Trp of BSA, the synchronous spectral measurements were achieved. The wavelength scanned was between 240 and 320 nm at temperature 297 K and pH 7.40.

#### 2.2.3. Fluorescence Resonance Energy Transfer (FRET)

The UV-Vis absorption spectrum and the emission fluorescence spectrum of RSN and BSA, respectively, with each concentration 3.0 × 10^−6^mol · L^−1^ were recorded in the wavelength range of 295–425 nm. Both the spectra were overlapped and Forster's nonradiative energy transfer theory was applied to calculate the distance between Trp134/Trp213 of BSA and RSN.

#### 2.2.4. Confirmation of Binding Site in the Presence of Site Markers

Warfarin, ibuprofen, and digitoxin are the three site markers for sites I, II, and III, respectively, used to study the competitive binding experiments. Fluorescence experiments of a BSA-RSN complex in the presence of site markers were carried out by adjusting the wavelengths at 290–420 nm, where the concentration of BSA and site markers is unchanged by keeping at 3.0 × 10^−6^mol · L^−1^, and the concentrations of RSN varied from 0.5 × 10^−6^ to 5.5 × 10^−6^mol · L^−1^ at 297 K and pH 7.40.

#### 2.2.5. Influence of Fe^2+^, Ni^2+^, Ca^2+^, Mg^2+^, and Cd^2+^ Ions on BSA-RSN Binding

The effect of Fe^2+^, Ni^2+^, Ca^2+^, Mg^2+^, and Cd^2+^ ions on the binding constant of BSA-RSN system was analyzed by recording the fluorescence spectra of BSA-RSN system in the presence of ions by keeping the concentration of BSA and metal ions constant at 3.0 × 10^−6^mol · L^−1^ with increasing the concentration of RSN from 0.5 × 10^−6^ to 5.5 × 10^−6^mol · L^−1^ in the wavelength range 290–420 nm at 297 K and pH 7.40.

#### 2.2.6. FT-IR Spectroscopy

*(1) Instrument*. All FT-IR spectral measurements were carried out on a FT-IR spectrometer (Spectrum Two, PerkinElmer, Waltham, MA 02451, USA). This instrument was equipped with a beam splitter, detector, and ATR (germanium attenuated total reflection) as OptKBr, deuterated-triglycine sulphate (MIRTGS), and MIRacle Diamond S2PE accessory, respectively, with about 90 scans and resolution of 4 cm^−1^.

*(2) Change in Conformation of BSA-RSN Interaction*. The FT-IR spectra were obtained at 297 K and pH 7.4 by keeping the concentration of BSA and RSN same (3.5 × 10^−6^mol · L^−1^). In addition to buffer background, the bound form of BSA with RSN was taken by correcting the buffer + (RSN-free form) background.

*(3) Alteration of Secondary Structure of BSA by RSN*. The curve fitting analysis enhanced by second derivative of self-deconvolution was applied to amide I band (1700–1600 cm^−1^) of FT-IR spectra in ORIGINPRO 9.0 64-bit software, where the overlapped peaks were characterized by single peak each corresponding to secondary structure of BSA after the interaction of RSN.

#### 2.2.7. Molecular Docking

Protein Data Bank (PDB) was used to download the crystal structure of BSA with PDB ID: 4F5S (https://www.rcsb.org/structure/4F5S). BSA was further subjected to Swiss-Pdb Viewer 4.1.0 software to minimize the energy to a very low extent by applying the GROMOS96 43B1 force field. The structure of risedronate sodium (RSN) was obtained from PubChem with CID 4194514 (https://pubchem.ncbi.nlm.nih.gov/compound/4194514). The energy minimization of RSN was done by using Marvin View 5.8.1. Now, the BSA and RSN were ready for molecular docking determinations. The software comprising Autodock tools, Autogrid 4.2, and Autodock 4.2 was employed to study the molecular docking for BSA-RSN binding interactions [20]. Chain B, residual atoms, heteroatoms, and water molecules were removed from BSA by keeping only chain A. Then, polar hydrogens and partial Kollman charges were added: nonpolar hydrogens were merged for BSA. The grid box of dimension 60 × 60 × 60 along *X*, *Y*, and *Z* axes with grid spacing 0.375 Å was adjusted. Then, the grid centered coordinates for site I (IIA), site II (IIIA), and site III (IB) were positioned at (−4.795, 30.487, and 101.007), (10.906, 16.276, and 119.720), and (19.857, 33.531, and 97.915), respectively, to analyze the molecular docking separately for each binding sites after interaction of BSA with RSN. The docking was run for all the three binding sites independently by running 90 times. After this process, the output was estimated using Lamarckian genetic algorithm (LGA) with maximum of 2,500,000 energy evaluations, 27,000 generations by assigning random translations, orientations, and 9 torsions to populations of 150 individuals. The obtained results were figured out by Biovia Discovery Studio 2016.

#### 2.2.8. Circular Dichroism (CD) Spectral Measurements

J-815 circular dichroism spectropolarimeter (Jasco international Co., Ltd., Tokyo, Japan) was used to obtain the CD spectra of free BSA and bound form of BSA with RSN. Furthermore, CD spectra were recorded in the region of 200–240 nm at 297 K with the cell length, bandwidth, and scanning speed of about 10 mm, 1.00 nm, and 100 nm/min, respectively. Baseline correction was also performed while noting down the CD spectra by buffer subtraction of pH 7.40. The ratio of the concentrations of BSA to RSN was kept at 1 : 20 (3.5 × 10^−6^ to 70 × 10^−6^mol · L^−1^). The secondary structure of BSA before and after interaction with RSN was found out by using the BeStSel software online server (bestsel.elte.hu/).

#### 2.2.9. Statistical Analysis and Curve Plotting

All the experiments were measured three times for accuracy, and the results were expressed in terms of mean  ±  standard deviation (SD). The acquired results were inspected and data were evaluated by OriginPro 9.0 64-bit software (OriginLab Corp., Northampton, MA) and Microsoft Excel.

## 3. Results and Discussion

### 3.1. UV-Vis Absorption of BSA with and without RSN

**Figure 2:**
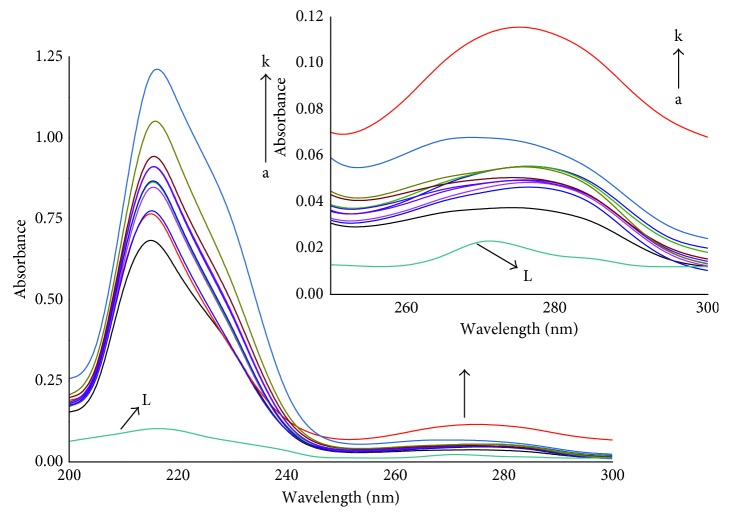
UV-Vis absorption spectra of BSA (2.5 × 10^−6^mol · L^−1^) with varying concentrations of RSN (0.5 to 5.5 × 10^−6^mol · L^−1^) with the addition of 0.5 × 10^−6^mol · L^−1^in each step, and the curve L represents RSN only (2.5 × 10^−6^mol · L^−1^) at 297 K and pH 7.40.

Change in the conformation of BSA and formation of the complex after the interaction with RSN is effectively studied by UV-Vis spectral measurements. The UV-Vis absorption spectrum of BSA with different concentrations of RSN (Figure 2) illustrates that the absorption intensity of BSA increases with increasing the concentration of RSN which confirms formation of the complex between BSA and RSN. Additionally, there are two absorption peaks with the red shifts (3 nm): the stronger one at 220 nm corresponding to the backbone of the BSA structure, and weaker one at 280 nm corresponding to the *π* → *π*^*∗*^ transition for tyrosine (Tyr), tryptophan (Trp), and Phenylalanine (Phe) residues of BSA as they belong to aromatic amino acids [21]. From the above, it concludes that RSN interacted with BSA and also this interaction affects microenvironment of Tyr, Trp, and Phe by altering conformation of BSA.

### 3.2. Fluorescence Measurements

**Figure 3:**
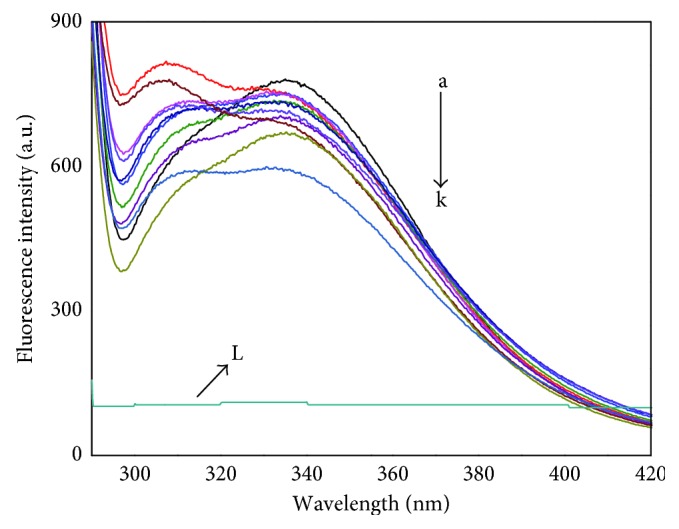
Fluorescence emission spectra of BSA (2.5 × 10^−6^mol · L^−1^) with varying concentrations of RSN (a to k: 0.5 to 5.5 × 10^−6^mol · L^−1^) with the addition of 0.5 × 10^−6^mol · L^−1^ in each step, and the curve L represents RSN only (2.5 × 10^−6^mol · L^−1^) at 297 K and pH 7.40.

Figure 3 shows the fluorescence emission spectra of BSA with the increasing concentration of RSN in the solution. It is clearly noticed from Figure 3 that the intensity of fluorescence for BSA decreases with increasing RSN concentration indicating that the RSN binds with BSA. Thus, BSA was quenched by RSN. Since the tyrosine and trypophan are the two aromatic amino acids, they are responsible for fluorescence emission. In this study, risedronic acid after interaction with BSA shows two emission peaks around one for tyrosine and the other for tryptophan at 308 and 335 nm, respectively, with a slight blue shift (3 nm) observed for tryptophan not for tyrosine. This spectral shift signifies the changes in microenvironment around tryptophan residues. Notably, the excitation occurs at 295 nm which is corresponding to tryptophan not for tyrosine (280 nm). Therefore, only tryptophan was involved in the interaction [22].

**Figure 4:**
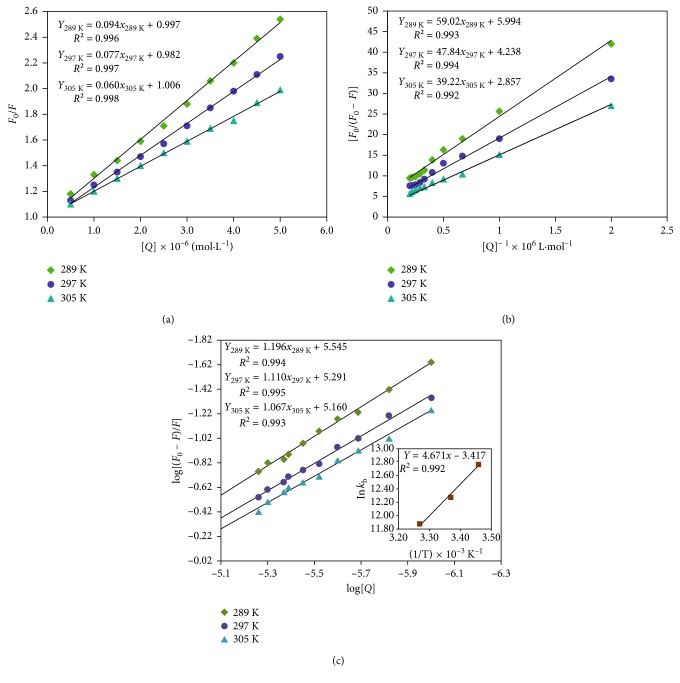
(a) Stern–Volmer plot, (b) modified Stern–Volmer plot, and (c) plot of log[(*F*_0_ − *F*)/*F*] versus log[*Q*] (the inset of (c) is van 't Hoff plot of the BSA-RSN complex) at different temperatures (289, 297, and 305 K) at pH 7.40.

Fluorescence quenching, molecular rearrangements, excited-state reactions, and energy transfer processes are consequences of interactions in the molecular level. Moreover, the decease of intensity of the fluorescence is called fluorescence quenching. There are two types of quenching mechanisms: dynamic and static. These two differ by dependence of quenching constant on temperature. The ground state complex formation defines the static quenching; molecules diffusing in the solution define dynamic quenching, where the quenching constant increases with the increase in temperature for dynamic quenching and vice versa for static quenching. The conventional Stern–Volmer equation (2) was used to examine the type of quenching mechanism [23].

(2) $$\begin{array}{c} \frac{F_{0}}{F}=1+k_{q}\tau_{0}\left[Q\right]=1+K_{\text{SV}}\left[Q\right], \end{array}$$

(3) $$\begin{array}{c} k_{q}=\frac{K_{\text{SV}}}{\tau_{0}}, \end{array}$$

where *F*_0_ and *F* are the fluorescence intensities of BSA and BSA-RSN bound form, respectively; [*Q*] and *τ*_0_ (∼5.7 × 10^−9^s) [24] are the concentration of RSN and fluorescence lifetime of biopolymer, respectively; and *K*_SV_ and *k*_q_ are the Stern–Volmer quenching constant and quenching rate constant, respectively.  Figure 4(a) represents the Stern–Volmer plot (*F*_0_/*F* versus [*Q*]), which gives the *K*_SV_ values as slope, and the values *k*_q_ are calculated by using (3). These are tabulated in Table 1 at different temperatures. Table 1 gives the information that both *K*_SV_ and *k*_q_ values decrease with increasing the temperatures. In addition to this, *k*_q_ values for dynamic quenching are 2 × 10^10^L · mol^−1^ · s^−1^, but in the present study, they are of the order 10^13^L · mol^−1^ · s^−1^ Hence, these results confirm that the quenching of BSA by RSN proceeds through static mechanisms. This static quenching mechanism of BSA by RSN was further confirmed by calculating the effective static quenching constant (*K*_a_) and accessible fraction of fluorophore (*f*_a_) in the BSA using the following modified Stern–Volmer equation (4) [25]:

(4) $$\begin{array}{c} \frac{F_{0}}{F_{0}-F}=\frac{1}{f_{a}}+\frac{1}{K_{a}f_{a}\left[Q\right]}. \end{array}$$

Figure 4(b) represents the modified Stern–Volmer plot, where the intercept and slope are equal to 1/*f*_a_ and 1/*K*_a_*f*_a_, respectively. Table 1 signifies that the obtained *K*_a_ values decrease with increasing temperature as do *K*_SV_ values. This confirms that RSN quenched the BSA by the static quenching mechanism.

### 3.3. Binding Parameters of RSN to BSA

The affinity of RSN binding with BSA can be obtained by calculating its binding constant (*K*_b_) and number of binding sites (*n*) using the following equation [26]:

(5) $$\begin{array}{c} \log\left[\frac{F_{0}-F}{F}\right]=\log{\;K}_{b}+n\;\log\left[Q\right]. \end{array}$$

The plot of log[(*F*_0_ − *F*)/*F*] versus log[*Q*] gives a straight line with slope equal to *n* and intercept equal to log*K*_b_ at different temperatures (289, 297, and 305 K) as presented in the Figure 4(c), and their values are tabulated in Table 2. The acquired *K*_b_ values decrease with increasing temperatures and of the order 10^5^, and they acknowledge that the strong binding exists between BSA and RSN and further confirm the static quenching mechanism. The *n* values are approximately equal to 1 signifying that only one RSN binds to BSA.

### 3.4. Thermodynamics and the Forces Involved in BSA-RSN Complex Stabilization

The noncovalent interactions between proteins and small molecules accomplished by determining the thermodynamic parameters, change in enthalpy (Δ*H*), and entropy (Δ*S*) mainly constitute electrostatic forces, hydrogen bonds, van der Waals force, and hydrophobic force. Δ*H* and Δ*S* are best related by the following van 't Hoff equation (6) [27]:

(6) $$\begin{array}{c} \ln\;K_{b}=\frac{-\Delta H}{RT}+\frac{\Delta S}{R}, \end{array}$$

where *R*, *T*, and *K*_b_ are the gas constant, temperature, and calculated binding constant. The values of Δ*H* and Δ*S* calculated by using the slope and intercept of the plot of ln *K*_b_ against 1/*T* are shown in inset of Figure 4(c). From Table 2, it is evident that the obtained negative values of Δ*H* and Δ*S* imitate the van der Waals forces and hydrogen bonding which are the main binding forces forming the BSA–RSN complex [28]. Then, these values are utilized to estimate Gibb's free energy which is connected by the following equation:

(7) $$\begin{array}{c} \Delta G=\Delta H-T\Delta S. \end{array}$$

The summarized Δ*G* values in Table 2 are negative and denote the spontaneous and exothermic process. Since the value of Δ*H* is less than Δ*S*, Gibb's free energy is typically entropy driven. Hence, the RSN bound to BSA dominates hydrogen bonding compared to van der Waals forces.

### 3.5. Synchronous Fluorescence Analysis

By simultaneously scanning excitation and emission monochromators in synchronous fluorescence spectral measurements, the micro-environmental changes of Tyr and Trp residues could be analysed. The change in polarity of Tyr and Trp residues' microenvironment is achieved by shift in *λ*_em_: red shift represents the increase of polarity/decrease of hydrophobicity, and blue shift represents decrease of polarity/increase of hydrophobicity [21].

**Figure 5:**
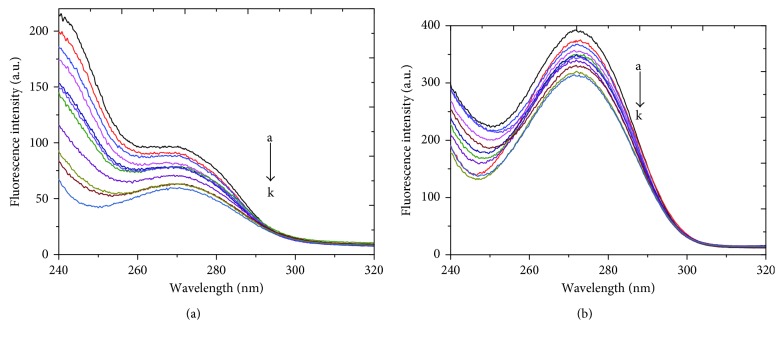
Synchronous fluorescence spectra of BSA (2.5 × 10^−6^ mol · L^−1^) after interaction with RSN (0 to 5.5 × 10^−6^ mol · L^−1^) at 297 K and pH 7.40: (a) Δ*λ* = 15 nm for Tyr and (b) Δ*λ* = 60 nm for Trp.

The synchronous fluorescence spectra for BSA with various concentrations of RSN at Δ*λ* = 15 and 60 nm for Tyr and Trp residues are as shown in Figure 5. There is no spectral shift observed for Tyr (Figure 5(a)), but a characteristic red shift of about 3 nm is observed for Trp (Figure 5(b)). Therefore, it is clear that the microenvironment of Trp occurs where it is more exposed to polar environment.

### 3.6. Energy Transfer between BSA and RSN

**Figure 6:**
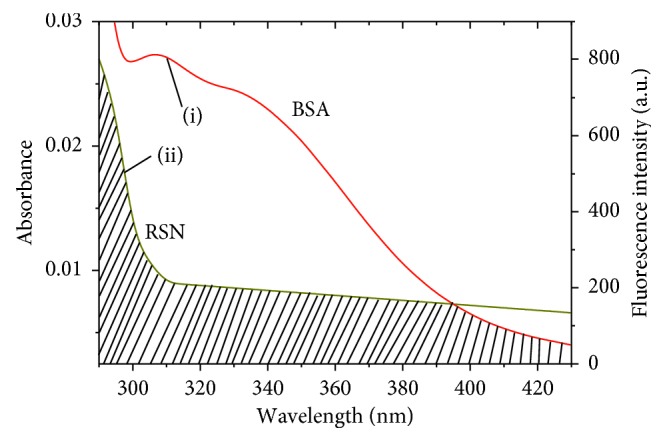
Overlap spectrum between (i) the fluorescence emission spectrum of BSA and (ii) the UV absorption spectrum of RSN. The concentration of BSA and RSN is 3.0 × 10^−6^mol · L^−1^ at 297 K and pH 7.40.

The transfer of excitation energy of various molecules which are already in excited states without emission of a photon from donor to acceptor has been used to find out ligand-Trp distances in protein. According to Forster's nonradiative energy transfer theory, fluorescence emission spectrum of donor and UV–vis absorption spectrum of the acceptor should overlap with their distance which lies between 1 to 10 nm. The energy transfer efficiency (*E*) can be written as

(8) $$\begin{array}{c} E=\frac{R_{0}^{6}}{R_{0}^{6}+r^{6}}=\frac{F_{0}-F}{F_{0}}, \end{array}$$

where *R*_0_ is the critical distance when *E* is 50%, and *r* is the mean distance between centers of the BSA and RSN dipoles. Then, the *R*_0_ (cm) can be represented by [23]

(9) $$\begin{array}{c} R_{0}^{6}=8.8\times 10^{-25}K^{2}N^{-4}ФJ, \end{array}$$

where *K*^2^ is spatial orientation factor of the dipole, *Ф* is fluorescence quantum yield of the BSA, *N* is the refractive index of the medium, and *J* is overlap integral of the fluorescence emission spectrum of BSA and absorption spectrum of the RSN (Figure 6). The value of *J* can be calculated by (10) at *λ* = 295–425 nm:

(10) $$\begin{array}{c} J=\frac{\sum \left[F_{d}\left(\lambda \right)\varepsilon_{a}\left(\lambda \right)\lambda^{4}\Delta \lambda \right]}{\sum \left[F_{d}\left(\lambda \right)\Delta \lambda \right]}, \end{array}$$

where *F*_d_ and *ε*_a_(*λ*) are the fluorescence intensity of the BSA at wavelength *λ* to *λ*+Δ*λ* (it has no unit) and the molar absorption coefficient of the RSN at wavelength is *λ*. In case of BSA, *K*^2^ = 2/3, *N* = 1.336, and *Ф* = 0.118. The obtained results are *J* = 1.19 × 10^−14^ cm^3^·L·mol^−1^, *E* = 70.44%, *r* = 0.267 Å, and *R*_0_ = 0.252 Å. Static quenching of RSN to BSA was further confirmed from the larger value of *r* compared to that of *R*_0_. BSA has only two tryptophan residues, that is, Trp134 and Trp213, mainly responsible for its fluorescence. So, the FRET tool allowed us for the determination of the distance between the Trp134/Trp-213 and bound RSN.

### 3.7. Competitive Binding Measurements

**Figure 7:**
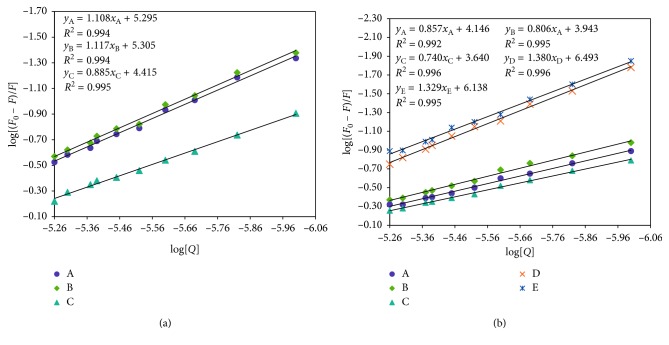
Plot of log[(*F*_0_ − *F*)/*F*] versus log[*Q*] for (a) site markers (digitoxin: A, warfarin: B, and ibuprofen: C) and (b) metal ions (Fe^2+^: A, Ca^2+^: B, Mg^2+^: C, Ni^2+^: D, and Cd^2+^: E) at 297 K and pH 7.40.

The determination of preferable binding site for RSN in BSA was achieved by performing competitive binding measurements. The three binding sites I, II, and III correspond to the subdomains IIA, IIIA, and IB, respectively. Warfarin, ibuprofen, and digitoxin categorically binds to site I, II, and III, respectively [29]. The binding constant values calculated for the BSA-RSN complex in the presence of all the site probes using (5) (Figure 7(a)) and values are tabulated in Table 3. From Table 3, it is understood that warfarin and digitoxin have no effect on BSA-RSN binding, but ibuprofen has a significant amount of effect where obtained binding constant value varies with a large difference. Hence, this result suggested that site II (IIIA) of BSA to undergo major binding with RSN. Further confirmations about the binding site were well understood from molecular docking studies.

### 3.8. Effect of Metal Ions on BSA-RSN Binding

Many chemical and biological processes in the human body are mediated via metal ions which show interaction with proteins. The fluorescence data of interaction between BSA and RSN in the presence and absence of Fe^2+^, Ni^2+^, Ca^2+^, Mg^2+^, and Cd^2+^ ions were subjected to binding calculations at 297 K using (5). The obtained values are charted in Table 3 and their plots are shown in Figure 7(b). From Table 3, it can be noticed that Fe^2+^, Ca^2+^, and Mg^2+^ ions decrease the binding between BSA and RSN, and Ni^2+^ and Cd^2+^ increase the binding between BSA and RSN. Ni^2+^ and Cd^2+^ ions increase the binding affinity of RSN and BSA due to the formation of metal ion-RSN complexes which in turn interact with the BSA through metal ion bridge. So, RSN remains interacting with BSA more where it enhances the effectiveness of RSN [30]. Decreased binding constants, in the presence of metal ions, may be due to the competition between ion and RSN and also due to the alternation of conformation of BSA. Therefore, as soon as RSN enters into the blood, it instantly comes out of it after interaction with BSA in the presence of Fe^2+^, Ca^2+^, and Mg^2+^ ions. So, short time of RSN in blood will not lead to curing of the required therapeutic effects. Thus, more dosage is required.

### 3.9. Conformational Changes of BSA by FT-IR Measurements

**Figure 8:**
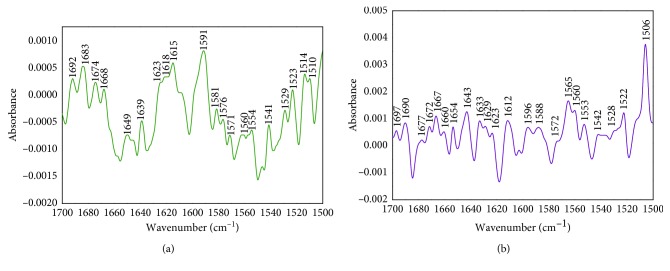
FT-IR spectra at 1700–1500 cm^−1^ at 297 K and pH 7.40. (a) Free BSA after subtraction from buffer and (b) bound BSA with RSN after subtraction from RSN and buffer. Concentration of BSA and RSN were kept constant at 3.5 × 10^−6^mol · L^−1^.

The protein exhibits a number of FT-IR bands, out of which amide bands give the vibrations of peptide linkage. There are two amide bands, I and II, which symbolize the characteristic conformational changes showing vibrations at 1700–1600 cm^−1^ (C=O stretch) and 1600–1500 cm^−1^ (combined stretching of C-N and bending of N-H), respectively.  Figure 8 represents the FT-IR spectra for BSA in the presence and absence of RSN, and their peak positions are tabulated in Table 4. The change in the spectral lines is observed from Figures 8(a) and 8(b), which reveal that the formation of a complex between BSA and RSN with conformational changes occurred in BSA.

### 3.10. Inspection of Secondary Structure of BSA

**Figure 9:**
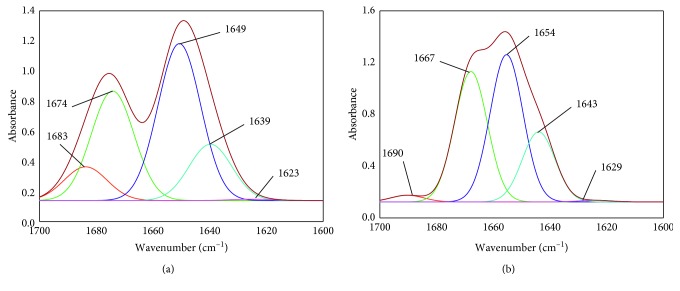
Amide I band (1700–1600 cm^−1^) of BSA (a) and BSA-RSN system (b) subjected to curve fitting analysis by second derivative resolution using Gaussian peak function.

The amide I band can be further analyzed for secondary structure of BSA as the bands are divided into *β*–sheets, random coil, *α*-helix, *β*-turns, and *β*-antiparallel, which correspond to the vibrations at 1615–1637, 1648–1648, 1649–1660, 1660–1680, and 1680–1692 cm^−1^, respectively [31].  Figure 9 shows that the amide I band is subjected to self-deconvolution by using curve-fitting procedures with second derivative resolution enhancement for BSA-RSN interaction, and the changes in the secondary structure conformation of BSA after interaction with RSN is tabulated in Table 5. The alteration of peak positions and amide I band shape gives an idea about BSA interacting with RSN by secondary structure of modified BSA.

### 3.11. Molecular Docking Analysis

**Figure 10:**
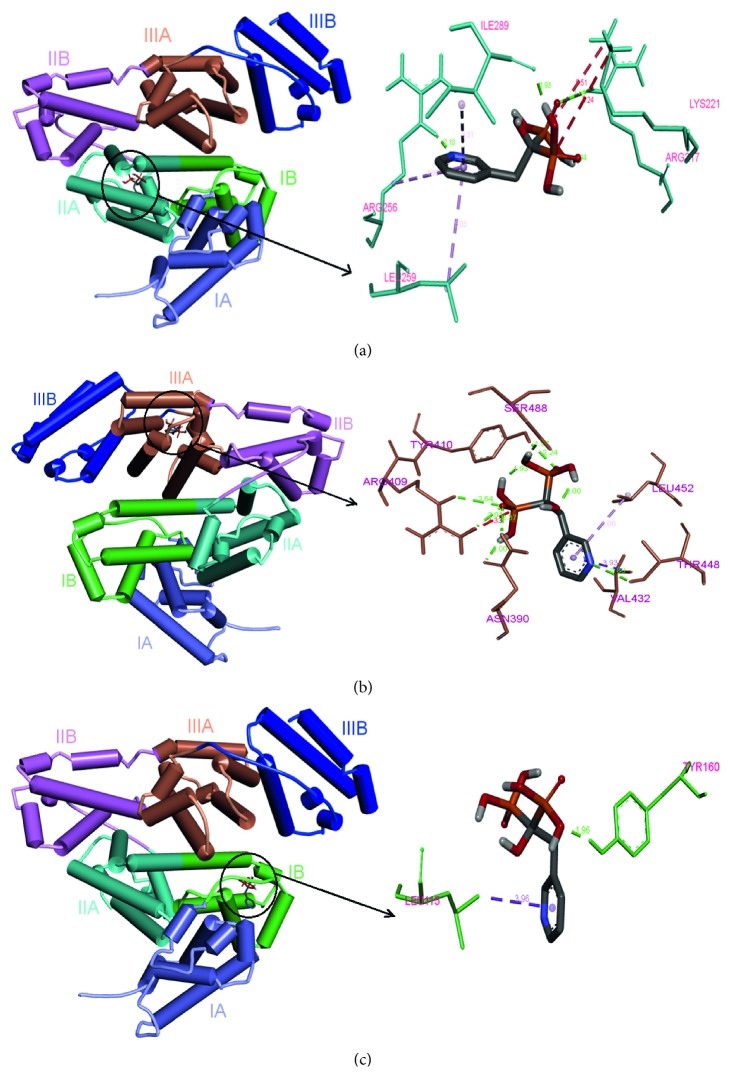
The molecular binding interaction of BSA and RSN for the three binding sites I (IIA), II (IIIA), and III (IB) by molecular docking studies.

The site marker experiment, thermodynamic calculations, and type of interaction from the fluorescence study of the BSA-RSN complex were further confirmed by molecular docking measurements which give all the major information about interaction studies. The ligand binding sites such as site I (IIA), II (IIIA), and III (IB) of BSA were separately docked with RSN. The cluster analysis by root mean square deviation (RMSD) respectively for 3 binding sites I, II, and III gives cluster modes (62, 89, and 35) and binding energy −25.03, −30.58, and −22.47 kJ·mol^−1^. Based on the lowest binding energy and largest cluster mode, site II (IIIA) of the BSA after interaction with RSN undergoes preferable binding compared to other two sites.  Figure 10 represents the docked results of each site where the amino acids surrounding the RSN could be clearly noticeable. In addition to this, interactions and their distances for all the binding sites are summarized in Table 6. Moreover, the results of site II (IIIA) were analyzed further.  Table 7 represents the top 5 ideal energy-ranked results of site II (IIIA) and the values Δ*G*, *E*_inter−mol_, *E*_vdw+HB+desol_, and *E*_Elec_ are the binding free energy; intermolecular interaction energy; sum of van der Waals energy, hydrogen bonding energy, and desolvation free energy; and electrostatic energy. More than 50% of the amino acid residues in site II surrounded by RSN belong to polar and ionic. It has been apparent from Table 7, *E*_vdw+HB+desol_ is observably more negative than *E*_Elec_, which signifies that hydrogen bonding and van der Waals forces are playing a crucial role in the complex formation between RSN and BSA, and this is in compliance with the thermodynamics result fetched from theoretical calculations.

### 3.12. Circular Dichroism (CD) Spectral Analysis

**Figure 11:**
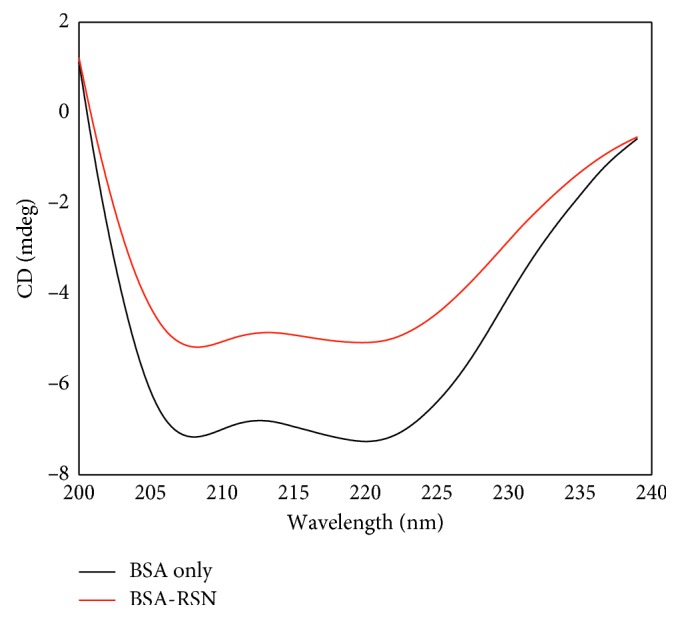
CD spectra of free BSA (black line) and BSA-RSN complex (red line) at pH 7.40 and 297 K. Concentration of BSA : RSN = 1 : 20 (3.5 × 10^−6^ to 70 × 10^−6^mol · L^−1^).

Circular dichroism (CD) spectral measurements are very effective tool in studying the conformational modifications of proteins upon interaction with small molecules or ligands. CD spectra of BSA in the absence and presence of RSN (shown in Figure 11) have two distinctive negative absorption bands at 208 nm (*π* → *π*^*∗*^ transition) and 222 nm (*n* → *π*^*∗*^ transition) corresponding to the *α*-helix of the peptide bond [32]. It could be noticed from Figure 11 that without shift in the band maxima, the two negative band intensities of free BSA were decreased after interaction with RSN, which induces the changes in the conformation of BSA with the decreasing *α*-helix content. The calculated secondary structures of BSA before and after interaction with RSN are tabulated in Table 8. The content of secondary structures (*α*-helix, *β*-strand antiparallel, and turn) of BSA after the interaction with RSN was decreased. But, the content of secondary structures (random coil and *β*-sheet parallel) of BSA after the interaction with RSN was increased. This represents that the polypeptide chain matrix of BSA was altered after interaction with RSN.

## 4. Conclusions

In the present work, the interaction between BSA and RSN were studied thoroughly by multi spectroscopic and molecular docking methods at physiological pH 7.40. RSN quenched the HSA by the static quenching mechanism. The formed HSA-RSN complex was stabilised by hydrogen bonding and van der Waals forces. The obtained binding constant value for HSA-RSN system is of the order 10^5^ signifying that strong binding exists between HSA and RSN. For HSA-RSN-Fe^2+^, HSA-RSN-Ca^2+^, and HSA-RSN-Mg^2+^ systems, the binding constants decreased. But for HSA-RSN-Cd^2+^ and HSA-RSN-Cd^2+^ systems, the binding constants increased. The subdomain IIIA, that is, site II, was the major binding site of BSA for RSN. The distance between Trp134/Trp213 of BSA and RSN was also achieved. The conformational and secondary structure of BSA was modified after the interaction with RSN. These types of studies are helpful to understand the metabolism, pharmacodynamics, and pharmacokinetics of RSN.

## Figures and Tables

**Table 1:** Stern–Volmer quenching constant, quenching rate constant, and effective static quenching constant for the BSA-RSN complex at different temperatures at pH 7.40.

Compound	Temperature (K)	*K*_SV_ (L · mol^−1^) × 10^4^	*k*_q_ (L · mol^−1^ · s^−1^) × 10^13^	*K*_a_ (L · mol^−1^) × 10^4^
BSA-RSN	289	9.4 ± 0.02	1.65 ± 0.01	10.16 ± 0.05
	297	7.7 ± 0.06	1.35 ± 0.03	8.86 ± 0.03
	305	6.0 ± 0.04	1.05 ± 0.02	7.28 ± 0.02

*K*_SV_ is the Stern–Volmer quenching constant; *k*_q_ is the quenching rate constant; *K*_a_ is the effective static quenching constant.

**Table 2:** Binding constants, number of binding sites, and thermodynamic parameters for the interaction of BSA with RSN at different temperatures (pH 7.40).

Compound	Temperature (K)	*K*_b_ (L · mol^−1^) × 10^5^	*n*	Δ*G* (kJ · mol^−1^)	Δ*H* (kJ · mol^−1^)	Δ*S* (J · mol^−1^ · K^−1^)
BSA-RSN	289	3.51 ± 0.10	1.196	−30.62	−38.83	−28.408
	297	1.95 ± 0.04	1.110	−30.39		
	305	1.45 ± 0.07	1.067	−30.16		

*K*_b_ is the binding constant; *n* is the number of binding sites; Δ*G* is the change in Gibb's free energy; Δ*H* is change in enthalpy; Δ*S* is change in entropy.

**Table 3:** The binding constant of the BSA-RSN system in the presence of site markers and metal ions at 297 K (pH 7.40).

System	*K*_b_ (L · mol^−1^)
BSA-RSN	1.95 ± 0.04 × 10^5^
BSA-RSN-warfarin	2.0 ± 0.02 × 10^5^
BSA-RSN-ibuprofen	2.6 ± 0.07 × 10^4^
BSA-RSN-digitoxin	1.97 ± 0.03 × 10^5^
BSA-RSN-Fe^2+^	1.39 ± 0.09 × 10^3^
BSA-RSN-Ni^2+^	3.11 ± 0.02 × 10^6^
BSA-RSN-Ca^2+^	8.77 ± 0.05 × 10^3^
BSA-RSN-Mg^2+^	4.36 ± 0.04 × 10^3^
BSA-RSN-Cd^2+^	1.30 ± 0.08 × 10^6^

*K*_b_ is the binding constant.

**Table 4:** FT-IR spectral position peaks of free BSA and after interaction with RSN at 297 K and pH 7.40.

System	Amide I (cm^−1^)					Amide II (cm^−1^)
	1615–1637	1638–1648	1649–1660	1660–1680	1680–1692	1548
Free BSA	1623 ± 0.42	1639 ± 0.95	1649 ± 0.34	1674 ± 0.67	1683 ± 0.41	1541 ± 0.29
BSA-RSN	1629 ± 0.84	1643 ± 0.62	1654 ± 0.56	1667 ± 0.85	1690 ± 0.72	1542 ± 0.38

**Table 5:** FT-IR spectral measurements of the secondary structure of BSA during interaction with RSN at 297 K and pH 7.40.

System	Secondary structure (%)				
	*β*-sheet	Random coil	*α*-helix	*β*-turn	*β*-antiparallel
Free BSA	0.39 ± 0.07	15.73 ± 0.02	43.86 ± 0.07	30.59 ± 0.04	9.42 ± 0.08
BSA-RSN	1.87 ± 0.02	36.63 ± 0.09	41.38 ± 0.05	19.69 ± 0.05	0.42 ± 0.03

**Table 6:** Type of interactions, involvement, and their distances between amino acids present in BSA after interaction with RSN for three binding sites by molecular docking studies.

Binding site	Interactions		Amino acid-RSN atom
	Type	Distance (Å)	
Site I (IIA)	Conventional hydrogen bond	2.10	ARG:256:HE–N1
		1.93	ILE289:O–H7
		2.16	ARG217:HE–O7
	van der Waals	—	SER286
		—	ILE263
		—	VAL292
	Carbon hydrogen bond	3.72	LYS221:CE–O7
	Unfavorable positive–positive	5.24	LYS221:NZ–P2
	Pi–alkyl	5.03	LEU259:(CG–CD1–D2)–aromatic ring
		5.31	ILE289:(CB–CG1–CD1)–aromatic ring
		4.95	ARG256: (CB–CG)–aromatic ring

Site II (IIIA)	Conventional hydrogen bond	2.64	ARG409:HE–O7
		2.00	ASN:OD1–H9
		2.37	ASN390:HD22–O7
		2.80	THR448:HG1–N1
		2.24	TYR410:HH–O6
		1.97	SER488:OG–H7
	van der Waals	—	PHE402
		—	LEU406
		—	LEU429
		—	LEU456
		—	LEU386
		—	ARG484
	Unfavorable positive–positive	3.33	ARG409:P1–NH2
	Pi–alkyl	5.06	LEU452:CG–aromatic ring

Site III (IB)	Pi–sigma	3.93	VAL432:CG1–aromatic ring
	Conventional hydrogen bond	1.96	TYR160:HH–O5
	van der Waals	—	LYS136
		—	PRO117
		—	GLU140
	Pi–sigma	3.96	LEU115:O1–aromatic ring

**Table 7:** Analysis of BSA docking with RSN at different conformations with Lamarckian genetic algorithm.

Rank	Run	Δ*G* (kJ · mol^−1^)	*E*_inter−mol_ (kJ · mol^−1^)	*E*_vdw+HB+desol_ (kJ · mol^−1^)	*E*_Elec_ (kJ · mol^−1^)
1	17	−30.79	−38.28	−37.07	−1.21
2	11	−29.49	−36.98	−36.27	−0.71
3	5	−29.37	−36.86	−36.10	−0.75
4	10	−29.03	−36.52	−36.35	−0.12
5	22	−28.11	−35.60	−35.60	+0.04

Δ*G* is the binding free energy; *E*_inter−mol_ is the intermolecular interaction energy; sum of van der Waals energy, hydrogen bonding energy, desolvation free energy, and electrostatic energy; *E*_vdw+HB+desol_ is the sum of van der Waals energy, hydrogen bonding energy, and desolvation free energy; *E*_Elec_ is the electrostatic energy.

**Table 8:** CD spectral results for estimating the percentage of the secondary structure of BSA before and after interaction with RSN.

Secondary structure (%)	Free BSA	BSA-RSN
*α*-helix	49.51 ± 0.52	40.06 ± 0.74
*β*-strand antiparallel	8.54 ± 0.46	5.91 ± 0.38
*β*-sheet parallel	0.57 ± 0.27	3.92 ± 0.22
Turn	25.06 ± 0.35	22.50 ± 0.77
Others (random)	16.30 ± 0.39	27.61 ± 0.27

## Data Availability

The data used to support the findings of this study are available from the corresponding author upon request.
